# SAR Image Generation Method Using DH-GAN for Automatic Target Recognition

**DOI:** 10.3390/s24020670

**Published:** 2024-01-20

**Authors:** Snyoll Oghim, Youngjae Kim, Hyochoong Bang, Deoksu Lim, Junyoung Ko

**Affiliations:** 1 Department of Aerospace Engineering, Korea Advanced Institute of Science and Technology, Daejeon 34141, Republic of Korea; s.oghim@kaist.ac.kr (S.O.); kim_yj2528@kaist.ac.kr (Y.K.); 2 Hanwha Systems, Yongin-si 17121, Republic of Korea; deoksu.lim@hanwha.com (D.L.); junyoung2658.ko@hanwha.com (J.K.)

**Keywords:** automatic target recognition, synthetic aperture radar, generative adversarial networks, convolutional neural networks

## Abstract

In recent years, target recognition technology for synthetic aperture radar (SAR) images has witnessed significant advancements, particularly with the development of convolutional neural networks (CNNs). However, acquiring SAR images requires significant resources, both in terms of time and cost. Moreover, due to the inherent properties of radar sensors, SAR images are often marred by speckle noise, a form of high-frequency noise. To address this issue, we introduce a Generative Adversarial Network (GAN) with a dual discriminator and high-frequency pass filter, named DH-GAN, specifically designed for generating simulated images. DH-GAN produces images that emulate the high-frequency characteristics of real SAR images. Through power spectral density (PSD) analysis and experiments, we demonstrate the validity of the DH-GAN approach. The experimental results show that not only do the SAR image generated using DH-GAN closely resemble the high-frequency component of real SAR images, but the proficiency of CNNs in target recognition, when trained with these simulated images, is also notably enhanced.

## 1. Introduction

In recent years, a dramatic surge in computing power has enabled the training of intricate neural network architectures, spurring advancements in machine learning. Among these deep learning models, convolutional neural networks (CNNs) are particularly noteworthy, as they can learn features directly from data, which enables them to identify and extract intricate patterns or objects from images with remarkable accuracy [1]. CNNs have shown remarkable potential, surpassing traditional image processing and analysis methods.

CNNs’ feature extraction capabilities have garnered considerable attention in target recognition for SAR images. While SAR images hold substantial commercial and military value, traditional target recognition methods face challenges due to the intrinsic noise and intricate patterns characteristic of these images. Along with research utilizing clustering techniques such as the support vector machine (SVM) for target recognition [2,3], the CNN-based approach has notably enhanced target recognition performance by addressing the inherent complexities in SAR images [4,5,6,7,8,9,10].

Nevertheless, there are several challenges in SAR-ATR using CNNs. A primary issue is that acquiring SAR images requires considerable resources and time, making securing enough samples for CNN training difficult. A lack of samples can potentially lead CNNs to overfit the training data, resulting in low recognition rates for new data not included in the training dataset.

Various studies have been conducted to address the scarcity of SAR images and improve SAR-ATR performance. One approach is data augmentation. This technique expands the dataset by applying simple transformations to the original data, such as translation, rotation, and brightness adjustment [11,12,13], while data augmentation can easily increase the sample size and the network’s generalization ability, its impact on performance improvement is limited. Another strategy involves generating simulated data for specific targets using 3D modeling [14]. This method can produce a range of SAR images that match user-defined characteristics. However, it necessitates a deep understanding of both SAR image and 3D modeling techniques. A further alternative involves leveraging data from other domains. For instance, transfer learning with infrared (IR) data, which are more accessible than SAR images, has been proposed as a viable strategy [15].

Recently, researchers have been utilizing various GAN models to generate and utilize simulated SAR images that resemble real SAR images. GANs learn real (training) dataset distributions through competition between two neural networks: a discriminator and a generator. Due to these characteristics, GANs are gaining attention as an innovative solution to address the sample scarcity of SAR images. The conditional GAN (cGAN), enhanced with conditional variables, was proposed to generate images with specific targets [16]. The deep convolutional GAN (DCGAN) based on semi-supervised learning was developed to enhance the quality of generated SAR images [17]. Cui et al. introduced a study employing the Wasserstein GAN (WGAN) for generating SAR images at specific azimuth angles [18]. Furthermore, a method employing Cycle-GAN has been suggested for the style transfer of images from simulated to real domains [19].

SAR images acquired using radar sensors inevitably contain speckle noise, a type of high-frequency noise. Despite this, many studies that use GANs to create simulated images overlook this noise. Consequently, images produced using GANs may look real when viewed in the spatial domain but are easily distinguishable in the frequency domain. This issue is because GAN models primarily utilize low-frequency components to distinguish between real and simulated data [20,21,22]. Although this characteristic can be robust to noise in general, given that speckle noise is a significant feature of SAR images, it is essential to properly consider high-frequency components in GAN models to achieve results similar to real SAR images.

This paper introduces a GAN model with a dual discriminator designed to enhance the high-frequency component of generated SAR images. The proposed Dual Discriminator with High-frequency Pass Filter GAN (DH-GAN) is built upon the foundation of the conditional DCGAN. The first discriminator differentiates between the simulated images produced by the generator and the real SAR images, similar to the discriminator in conventional GANs. The newly added second discriminator utilizes the high-frequency components of the image to distinguish between real SAR images and simulated images. The generator is trained to deceive both discriminators simultaneously, generating simulated images that emulate the high-frequency traits of real SAR images more accurately.

The contributions of this paper are as follows: First, the proposed method can generate SAR images with high-frequency components similar to real SAR images. This is significant in that it considers the speckle noise characteristics of real SAR images, which existing GAN models have overlooked. Second, it is possible to generate high-quality labeled data by designing a GAN model based on the structure of cGAN and DCGAN. This is an important advantage as CNN training datasets require accurate labels. Finally, using simulated images created using the DH-GAN model allows for efficiently mitigating the overfitting issue in ATR-CNN. Notably, the high-frequency components in simulated SAR images closely resemble those in authentic SAR images, significantly enhancing the generalization capability of CNNs and recognition accuracy for SAR-ATR. The proposed DH-GAN is validated through various experiments.

## 2. Methods

### 2.1. Theoretical Background

**Figure 1:**
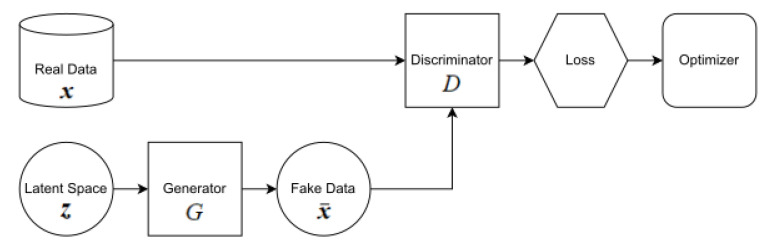
Basic structure of a GAN.

The GAN framework involves a competitive process between a discriminator (*D*) and a generator (*G*) to simulate data. Given a latent vector *z* drawn from the probability distribution $p_{z}$ in the latent space, the generator produces simulated data. Simultaneously, the discriminator aims to differentiate between real and simulated data sampled from the data distribution $p_{data}$. The neural network parameters for the generator and discriminator are iteratively updated using various loss functions. The basic architecture of a GAN is depicted in Figure 1.

Here, *x* denotes the real SAR image sampled from real data and $\bar{x}=G\left(z\right)$ represents the simulated image produced by the generator.

The original loss function of the GAN effectively categorizes the model into minimax games [23]; however, there can be saturation in the generator’s loss function during practical implementations. If the learning rate of the generator does not keep pace with that of the discriminator, the latter tends to dominate. This can lead to the premature termination of training, preventing the generator from learning effectively. To address this challenge, the Non-saturating GAN (NSGAN) was proposed, which adjusts the generator’s loss function as follows:

(1a) $$L_{D}^{NSGAN}=-E_{x∼p_{data}}\left[\log D\left(x\right)\right]-E_{z∼p_{z}}\left[\log\left(1-D\left(G\left(z\right)\right)\right)\right]$$

(1b) $$L_{G}^{NSGAN}=-E_{z∼p_{z}}\left[\log D\left(G\left(z\right)\right)\right]$$

A limitation of using NSGAN for SAR-ATR is the challenge in discerning the specific target represented by the simulated images. This is attributed to the inherent complexity of SAR images, which typically require specialized knowledge for visual interpretation. The conditional GAN (cGAN) offers a solution to this problem. cGAN extends the basic GAN architecture by incorporating condition variables [24]. Its primary objective is to produce data in alignment with given conditions. In this context, if the condition is set based on the target label, the cGAN generates an image corresponding to that label. Consequently, the user can obtain a SAR image of the desired target.

### 2.2. DH-GAN Model Framework

**Figure 2:**
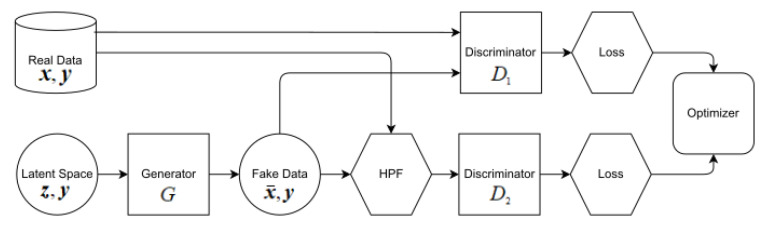
The structure of DH-GAN.

In this study, we propose a new model called DH-GAN. This model aims to generate simulated SAR images reflecting the high-frequency components of real SAR images. For this purpose, we have augmented the basic NSGAN with an additional discriminator neural network. The first discriminator distinguishes between real and simulated data. The second discriminator also distinguishes between real and simulated data, where both are emphasized with high-frequency components via HPF. The generator is trained to fool both discriminators, thereby ensuring that it generates images reflecting the inherent high-frequency characteristics of real images. Note that the inputs to all neural networks contain conditional parameters, similar to cGAN, to ensure that the generator can produce the desired target data. The architecture of the proposed DH-GAN is illustrated in Figure 2.

Here, *y* represents the label of the target, serving as the condition, and $D_{2}$ denotes the newly introduced discriminator. The real SAR images and simulated images, filtered using HPF, are used as input to $D_{2}$. The loss functions for the two discriminators and the generator are defined as follows:

(2a) $$L_{D_{1}}^{DH-GAN}=-E_{x∼p_{data}}\left[\log D_{1}\left(x|y\right)\right]-E_{z∼p_{z}}\left[\log\left(1-D_{1}\left(G\left(z|y\right)\right)\right)\right]$$

(2b) $$L_{D_{2}}^{DH-GAN}=-E_{x∼p_{data}}\left[\log D_{2}\left(f\left(x|y\right)\right)\right]-E_{z∼p_{z}}\left[\log\left(1-D_{2}\left(f\left(G\left(z|y\right)\right)\right)\right)\right]$$

(2c) $$L_{G}^{DH-GAN}=-\frac{1}{2}E_{z∼p_{z}}\left[\log D_{1}\left(G\left(z|y\right)\right)\right]-\frac{1}{2}E_{z∼p_{z}}\left[\log D_{2}\left(f\left(G\left(z|y\right)\right)\right)\right]$$

For a given image *A*, the function $f\left(A\right)$ is defined as the convolution of *A* with the kernel $K_{HPF}$, which represents a user-defined kernel.

For training the first discriminator, we employ the loss function given by Equation (2a). Meanwhile, the second discriminator is trained using the loss function in Equation (2b). We take the average of the resulting losses from both discriminators to train the generator, as shown in Equation (2c).

**Figure 3:**
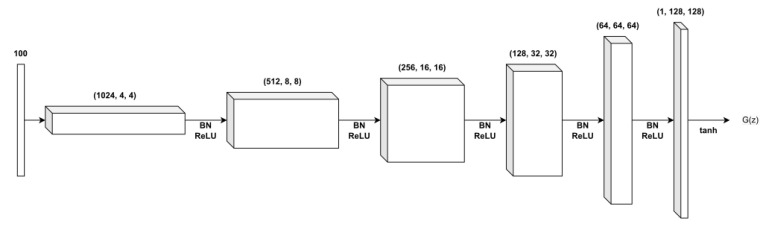
The network structure of the generator.

**Figure 4:**
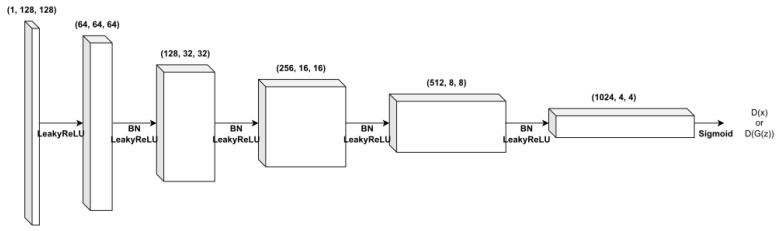
The network structure of the two discriminators.

In this study, the neural network structure of DH-GAN is founded on the DCGAN, which comprise a convolutional layer, batch normalization, and the ReLU activation function. Figure 3 shows the structure of the generator’s neural network. The generator can take as input a latent vector *z* of size 100 and eventually produce a simulated image of size 128×128. Here, the latent vector is drawn from the normal distribution. Figure 4 shows the structure of the discriminator’s neural network. The discriminator takes a 128×128 SAR image as input and outputs the probability that the input image is a real SAR image. Thus, the input data can be either a real SAR image or a simulated image from the generator. Note that both discriminators in DH-GAN have the same structure.

### 2.3. Power Spectrum Density Analysis

There exist several methods to assess simulated data produced using GANs [25,26,27,28]. Given that this paper aims to generate simulated data reflecting the high-frequency components of the original dataset, we employ power spectral density (PSD) analysis. PSD leverages the Fourier transform to depict the distribution of power across the frequency domain of an image. The image’s Fourier transform is expressed as follows:

(3) Fu,v=1WH∑x=0W−1∑y=0H−1fx,ye−j2πuxuxWW+vyvyHH

where *u* and *v* are coordinates of spatial frequencies, and *W* and *H* are the width and height of the image. The PSD of an image is calculated as follows:

(4) $$\mathrm{PSD}\left(u,v\right)={\left|F\left(u,v\right)\right|}^{2}$$

For the PSD analysis of images, the PSD is expressed as a function of wavenumber. The wavenumber is defined as a distance in the frequency domain; thus, it corresponds to the Euclidean distance from the center of the image to each pixel, $\omega_{k}=\sqrt{u^{2}+v^{2}}$. Due to the circular symmetry of the PSD, calculating the average PSD with respect to the wavenumbers necessitates averaging all the PSD values equidistant from the center of the image.

(5) $$\mathrm{PSD}\left(\omega_{k}\right)=\frac{1}{N\left(\omega_{k}\right)}\sum_{u,v}\mathrm{PSD}\left(u,v\right)$$

A high PSD value means the image has substantial energy at that particular frequency. Specifically, a raised PSD value at a higher wavenumber indicates the image’s inclusion of complex patterns or high-frequency components, such as edges or noise. Conversely, elevated PSD values in regions with lower wavenumber point to smoother sections or low-frequency components within the image.

### 2.4. CNN Model Frame

We employed a straightforward and shallow CNN model for the SAR-ATR [4]. Given that there is no unique structure or procedure exclusive to SAR images, the impact of the proposed DH-GAN can be distinctly evaluated. The CNN model directly ingests SAR images as input. Features from the SAR image are extracted via the CNN and subsequently channeled to a softmax activation function for classification. The CNN model adopted in this research is summarized in Table 1.

## 3. Experimental Study

### 3.1. MSTAR Dataset

We trained the DH-GAN and CNN models using the MSTAR dataset, released by DARPA (Defense Advanced Research Project Agency) for benchmarking ATR algorithms. The MSTAR dataset comprises SAR images representing 10 different ground targets. For each target, SAR images have been acquired from various depression and azimuth angles. Table 2 shows the number of SAR images of 10 targets for the two depression angles of 15 deg and 17 deg.

The experiment was conducted in two parts. The first part involved training a GAN model using the real dataset, and then using the trained generator to produce simulated images for 10 ground targets. The second part consisted of training the CNN and evaluating its recognition accuracy. In this section, we compare the results of training the CNN exclusively with the MSTAR dataset against those obtained when the training included the simulated dataset. These experimental procedures were also conducted for NSGAN and LSGAN for a comparative analysis with the DH-GAN results. Here, the real dataset refers to the MSTAR dataset, and the simulated dataset refers to the dataset generated by the GAN models.

### 3.2. SAR Image Generation

For CNN training, we utilized SAR images with a depression angle of 17 degrees. Meanwhile, to assess the recognition rate of the trained CNN, images with a depression angle of 15 degrees were employed. Consequently, to ensure compatibility and consistency, the DH-GAN was also trained using images with a depression angle of 17 degrees, enabling it to generate SAR images at the same depression angle.

Approximately 2500 original images with a depression angle of 17 degrees are insufficient for training the DH-GAN. To address this sample scarcity, we employed three data augmentation techniques. By flipping, rotating, and brightening the original SAR images, we generated 3000 images per target. In total, this yielded around 32,500 images for training the DH-GAN.

Given that the generator employs the tanh activation function, the input SAR images were scaled to fall within the [−1, 1] range. This adjustment enhanced the training speed and stability by aligning the input and output ranges. Both the discriminators and the generator utilized the Adam optimizer, with optimizer parameters $\beta_{1}$ and $\beta_{2}$ set to 0.5 and 0.999, respectively. The learning rate was configured at 0.00002, the batch size was 16, and the training was performed for 10 epochs.

The input for the second discriminator requires SAR images with emphasized high-frequency components. To achieve this, we introduced a preprocessing step that accentuated these high-frequency elements in the SAR image. We employed a high-frequency pass filter (HPF) for this purpose, with the kernel $K_{HPF}$ defined as follows:

(6) $$K_{HPF}=\left[\begin{array}{ccc} -1 & -1 & -1\\ -1 & 8 & -1\\ -1 & -1 & -1 \end{array}\right]$$

**Figure 5:**
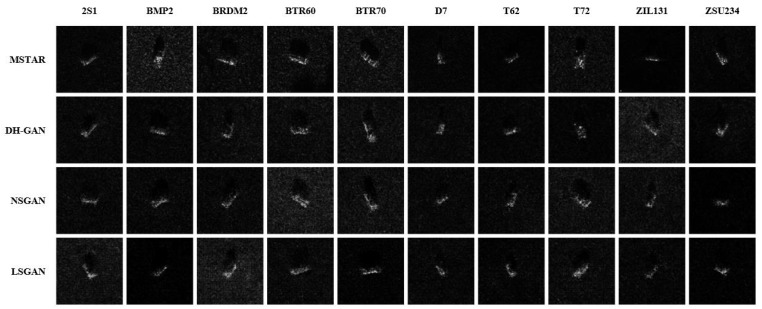
The result of DH-GAN, NSGAN, and LSGAN at epoch 10.

Figure 5 displays the simulated images produced using DH-GAN, as well as those generated using NSGAN and LSGAN for comparison. Both NSGAN and LSGAN are built upon DCGAN with a conditional parameter. The sole distinction between NSGAN, LSGAN, and DH-GAN lies in the absence of a second discriminator and HPF for NSGAN and LSGAN. Furthermore, the training conditions and datasets for NSGAN and LSGAN align with those used for DH-GAN.

**Figure 6:**
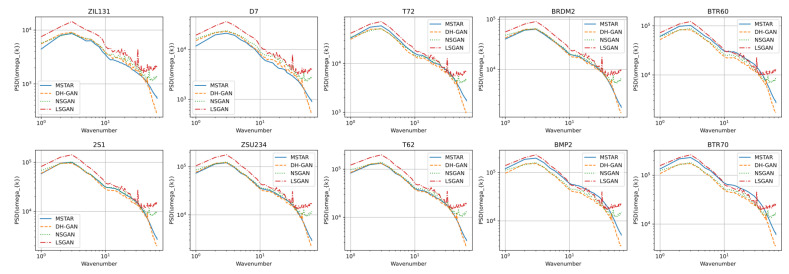
Power spectrum density analysis for simulated datasets.

When assessing the simulated datasets from a human perspective, all three models produced results similar to real images, and differentiating them became challenging. However, this parity was disrupted through power spectrum density (PSD) analysis. As depicted in Figure 6, the PSD of real datasets and those generated using the three models differed considerably. The real dataset’s PSD, represented by a solid blue line, indicated that, on a log scale, the PSD decreased as frequency increased. In contrast, the results for NSGAN and LSGAN, illustrated with green dotted and red dash–dot lines, respectively, showed higher distributions beyond a specific frequency. These results occur because the discriminators of LSGAN and NSGAN mainly distinguish between real SAR images and simulated images based on low-frequency features, such as structural or morphological aspects, of the images.

Conversely, the PSD of the dataset generated by DH-GAN, depicted as an orange dotted line, closely resembles the spectrum of the real data. This similarity indicates that the second discriminator—trained specifically on images with emphasized high-frequency components—effectively induce the generator in producing images that reflect the high-frequency components of actual data. This demonstrates that the proposed dual discriminator structure and loss function are functioning effectively as intended.

### 3.3. Recognition Performance

We subsequently assessed the recognition rate performance of the CNN model for SAR-ATR using the previously created simulated dataset. For training, the CNN utilized the original dataset, which has a depression angle of 17 degrees, along with the simulated datasets. Three distinct simulated datasets were available, generated using DH-GAN, NSGAN, and LSGAN, each containing 1000 images per target. Throughout the CNN training phase, all 10 targets served as inputs, with each input SAR image having a resolution of 128 × 128. The CNN model training employed Stochastic Gradient Descent (SGD) with a learning rate of 0.0125, a momentum coefficient 0.9, and a weight decay coefficient of $5\times 10^{-5}$. The training was conducted over 100 epochs with a batch size of 64.

**Figure 7:**
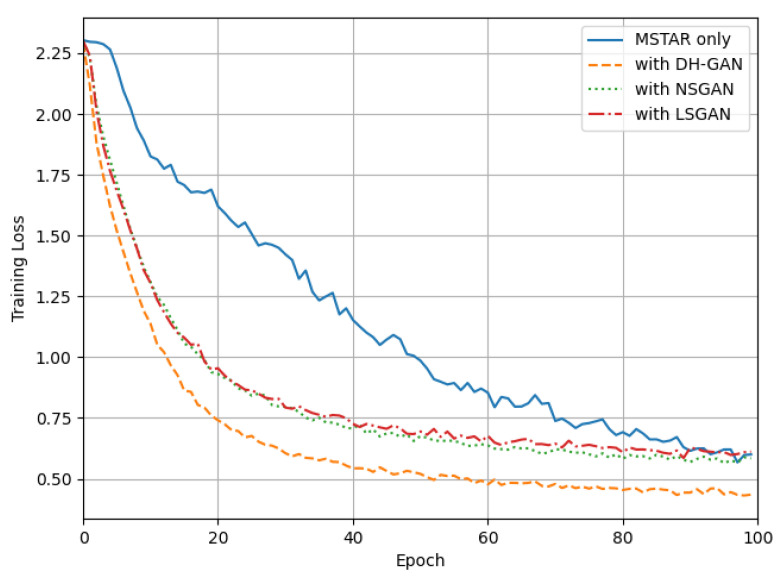
The training loss graph.

**Figure 8:**
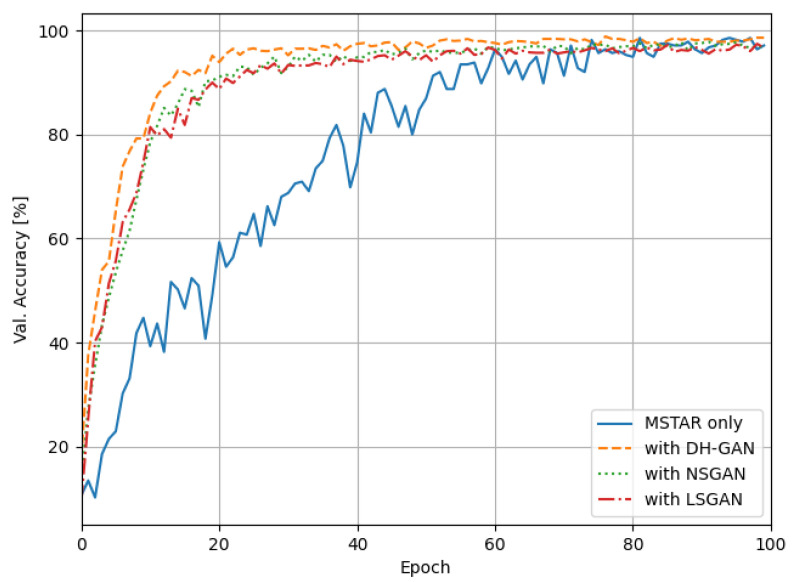
The recognition rate graph.

Figure 7 displays the CNN traning loss graph for each simulated dataset. As the training results in Figure 8 indicate, the use of simulated datasets accelerated the training process, and the training loss was lower compared to using only the MSTAR data. Among these datasets, the data generated by DH-GAN exhibited the lowest training loss and slightly outpaced the training speed of both NSGAN and LSGAN. These results suggest that the dataset generated by DH-GAN more effectively addresses the issue of insufficient training data compared to those generated by other GAN models. In other words, the data generated by our proposed method demonstrates characteristics more closely resembling real data.

Next, we assessed the recognition performance of each CNN model using a SAR image with a depression angle of 15 degrees, which was not included in the training dataset. Table 3 summarizing the average recognition rate for 10 targets shows that the CNN model’s recognition performance is enhanced when utilizing the simulated datasets in conjunction with the MSTAR. In particular, the recognition rate showed a slight improvement with the DH-GAN dataset compared to those of the other two GAN models. While this improvement in recognition rate might appear modest, it offers a significant advantage by achieving high recognition performance in fewer training epochs.

These experimental results demonstrate the effectiveness of the proposed DH-GAN. The second discriminator with HPF and loss functions in Equation (2) are instrumental in aiding the generator to produce more realistic images by reflecting the high-frequency components of real images. Notably, the dataset generated by DH-GAN enhances the training speed and recognition performance of CNN to a greater extent than those generated by other GAN models. This results indirectly highlights the significance of high-frequency components in the real dataset, something that is often overlooked by existing GAN models.

## 4. Conclusions

The advent of CNNs has brought about remarkable advancements in target recognition for SAR images. Nevertheless, the broader application of CNNs is limited by challenges in acquiring ample SAR images. To solve this sample scarcity, GAN-based methods for generating simulated images have been proposed. However, when these images are assessed in the frequency domain, there is a clear distinction between the real and simulated images. To bridge this gap, we present the DH-GAN. This model is designed to generate simulated images that emulate the high-frequency characteristics intrinsic to real SAR images. The structure of the proposed DH-GAN is based on a conditional GAN with convolutional neural network, and a discriminator equipped with a high-pass frequency filter is added.

Through PSD analysis, we are able to show that the images generated using DH-GAN closely resemble the inherent high-frequency characteristics in real SAR images. By incorporating these synthetic SAR images into the training dataset, the target recognition capabilities of the CNN were notably enhanced. Moreover, when compared to datasets produced using NSGAN and LSGAN, the validity of the DH-GAN approach became evident. Our proposed approach can generate more realistic SAR images for specific targets. This approach shows promise in enhancing recognition performance and addressing the overfitting challenges commonly faced by CNNs in SAR-ATR applications.

In future work, we plan to utilize not only PSD analysis but also explore new ways to evaluate the simulated images. In addition, we will simulate data that reflect various characteristics in addition to the high-frequency components of the real data, and analyze the impact of specific components of the SAR image on the recognition rate of the CNN. This research will be applied to various SAR imagery beyond the MSTAR dataset to propose data generation methods in the field and improve the recognition rate of CNNs. The final goal is to develop a CNN with robust recognition rates that can be applied to wide-area SAR images containing multiple targets acquired under mission scenarios. Moreover, the implications of DH-GAN’s enhanced accuracy in SAR image generation can be extended beyond military applications to disaster response and environmental monitoring. Future research could explore the integration of DH-GAN with satellite imaging technologies for real-time global monitoring capabilities.

## Figures and Tables

**Table 1:** The CNN structure for SAR-ATR.

Layer Type	Image Size	Feature Maps	Kernel Size	Function
Input	128 × 128	1	-	-
Convolution	120 × 120	18	9 × 9	ReLU
Pooling	20 × 20	18	6 × 6	Max Pooling
Convolution	16 × 16	36	5 × 5	ReLU
Pooling	4 × 4	36	4 × 4	Max Pooling
Convolution	1 × 1	120	4 × 4	ReLU
Fully Connected	-	1	120 × 10	Softmax
Output	10	-	-	-

**Table 2:** MSTAR datasets used for training and validation.

Target Name	Depression Angle = 17 deg	Depression Angle = 15 deg
2S1	299	274
BMP2	232	195
BRDM2	298	274
BTR60	256	195
BTR70	233	196
D7	299	274
T62	299	273
T72	232	196
ZIL131	299	274
ZSU234	299	274

**Table 3:** The recognition rate results of CNNs trained with different simulated datasets.

Name	MSTAR Only	With DH-GAN	With NSGAN	With LSGAN
2S1	88.32	94.53	91.61	94.16
BMP2	80.51	89.23	93.33	93.85
BRDM2	93.07	91.97	98.54	89.78
BTR60	96.41	97.44	94.36	93.85
BTR70	92.86	98.47	97.96	97.96
D7	98.91	98.54	99.27	98.91
T62	92.31	97.80	91.94	97.07
T72	98.47	95.41	92.86	95.92
ZIL131	98.18	95.62	96.35	95.26
ZSU234	98.18	99.27	97.45	97.81
Avg.	93.73	95.83	95.37	95.46

## Data Availability

Data are contained within the article.
